# Use of molecular modeling and site-directed mutagenesis to define the structural basis for the immune response to carbohydrate xenoantigens

**DOI:** 10.1186/1471-2172-8-3

**Published:** 2007-03-12

**Authors:** Mary Kearns-Jonker, Natasha Barteneva, Robert Mencel, Namath Hussain, Irina Shulkin, Alan Xu, Margaret Yew, Donald V Cramer

**Affiliations:** 1 Department of Cardiothoracic Surgery, Saban Research Institute of the Children's Hospital of Los Angeles, University of Southern California Keck School of Medicine, 4650 Sunset Blvd, Mailstop #137, Los Angeles, CA 90027 USA

## Abstract

**Background:**

Natural antibodies directed at carbohydrates reject porcine xenografts. They are initially expressed in germline configuration and are encoded by a small number of structurally-related germline progenitors. The transplantation of genetically-modified pig organs prevents hyperacute rejection, but delayed graft rejection still occurs, partly due to humoral responses. IgV_H _genes encoding induced xenoantibodies are predominantly, not exclusively, derived from germline progenitors in the V_H_3 family. We have previously identified the immunoglobulin heavy chain genes encoding V_H_3 xenoantibodies in patients and primates. In this manuscript, we complete the structural analysis of induced xenoantibodies by identifying the IgV_H _genes encoding the small proportion of V_H_4 xenoantibodies and the germline progenitors encoding xenoantibody light chains. This information has been used to define the xenoantibody/carbohydrate binding site using computer-simulated modeling.

**Results:**

The VH4-59 gene encodes antibodies in the V_H_4 family that are induced in human patients mounting active xenoantibody responses. The light chain of xenoantibodies is encoded by DPK5 and HSIGKV134. The structural information obtained by sequencing analysis was used to create computer-simulated models. Key contact sites for xenoantibody/carbohydrate interaction for V_H_3 family xenoantibodies include amino acids in sites 31, 33, 50, 57, 58 and the CDR3 region of the IgV_H _gene. Site-directed mutagenesis indicates that mutations in predicted contact sites alter binding to carbohydrate xenoantigens. Computer-simulated modeling suggests that the CDR3 region directly influences binding.

**Conclusion:**

Xenoantibodies induced during early and delayed xenograft responses are predominantly encoded by genes in the V_H_3 family, with a small proportion encoded by V_H_4 germline progenitors. This restricted group can be identified by the unique canonical structure of the light chain, heavy chain and CDR3. Computer-simulated models depict this structure with accuracy, as confirmed by site-directed mutagenesis. Computer-simulated drug design using computer-simulated models may now be applied to develop new drugs that may enhance the survival of xenografted organs.

## Background

Xenoantibodies represent a subset of natural antibodies that are present in normal humans as a consequence of our natural immune defense to infectious agents. These antibodies are evolutionarily conserved and can be classified into small groups on the basis of binding specificity and canonical structure [1,2]. The majority of xenoantibodies that initiate the rejection of porcine xenografts obtained from wild-type donors are specific for the α-gal epitope (Gal α1-3Galβ1-4GlcNAc-R) [3,4]. In normal humans, non-human primates and galactosyltransferase knockout mice, these antibodies are encoded by a small number of germline genes [5-10]. The availability and design of genetically-engineered donor organs has more recently led to the opportunity to study humoral responses that initiate acute xenograft rejection. Xenoantibodies mediating this process in response to placement of hDAF-transgenic organs are encoded by germline progenitors that are identical to those encoding xenoantibodies induced in response to wild-type pig organs, albeit at a delayed tempo [6,9,10,27]. In humans and non-human primates, these germline progenitors are alleles of IGHV3-11 [6,9,10]. In galactosyltransferase-deficient mice, a clonal expansion of antibodies encoded by the germline progenitor V_H_J606 occurs following graft placement [8]. The molecular basis for this restriction is not understood.

Genetically-modified donor organs do not induce hyperacute rejection, but acute vascular rejection still occurs [11-16]. The possibility that alternative carbohydrates may be relevant targets of induced antibody responses to genetically-modified xenografts has raised an interest in the structure and functional characteristics of anti-carbohydrate antibodies [13-19]. Natural xenoantibodies are encoded by a small number of germline progenitors with affinities for the gal carbohydrate that vary substantially [20-23]. The avidity of these antibodies for the gal carbohydrate does not increase following perfusion through porcine livers [20,21] but xenoantibodies produced in patients exposed to porcine islets do demonstrate an increase in affinity for the gal carbohydrate over time [22]. Natural antibodies and induced xenoantibodies in baboons do not differ in affinity for the gal carbohydrate [23], but rodent monoclonal anti-carbohydrate antibodies with mutations and a higher affinity for the gal carbohydrate can reject xenografts at an accelerated tempo [24]. The association between xenoantibody structure, somatic mutation and function during the progression of the immune response, *in vivo*, remains unclear. It has been postulated that the canonical structure of the germline progenitor plays a key role in the specificity of select antibodies for carbohydrates [25]. High resolution crystallography has shown that anti-carbohydrate antibodies in germline configuration can recognize a range of structurally-related carbohydrate epitopes [26]. Antibodies that bind to gal and structurally-related non-gal carbohydrates may therefore be encoded by the same group of germline progenitors. The relevance of this to the xenoantibody response is that accurate structural information defining xenoantibodies may be used to identify small molecular inhibitors that target anti-carbohydrate xenoantibodies and may enhance xenograft survival in multiple settings in which induced antibody-mediated responses prevent long-term graft survival.

Our laboratory has defined a select, restricted usage of IgV_H _genes that encode xenoantibodies in multiple settings. These antibodies have a unique canonical structure that is conserved across species [6,9,10,27]. By performing a detailed analysis of the sequence of IgV_H _genes encoding xenoantibodies expressed in patients and large animals mounting active immune responses to pig cells, an understanding of the three dimensional configuration of the xenoantibody/gal binding site can now be determined. In addition, sites of recurring mutations *in vivo *can be placed in context of carbohydrate/xenoantibody binding sites that can be predicted using computer-simulated models. This information provides a novel and important link in the development of reagents that can block anti-carbohydrate xenoantibodies that induce xenograft rejection.

In this manuscript, the structural basis of carbohydrate/xenoantibody interaction has been defined by sequence information comparing germline and mutated xenoantibodies. A computer-simulated model has been generated to define the unique structure of induced xenoantibodies in humans and non-human primates. Specific sites relevant for optimal xenoantibody/carbohydrate interaction have been identified and the role of somatic mutation *in vivo *at these sites during induced antibody responses can now be studied to provide insight on the role of mutations in the progression of the xenoantibody response. Our data suggest that the configuration of the xenoantibody binding pocket of the small group of IgM antibodies expanded during hyperacute and acute rejection responses is similar. Five contact sites within the heavy chain and the CDR3 region play a role in optimal xenoantibody/gal carbohydrate binding.

## Results

### Exposure of patients to pig cells stimulates an increase in the expression of antibodies encoded by the V_H_4-59 germline progenitor

**Figure 1:**
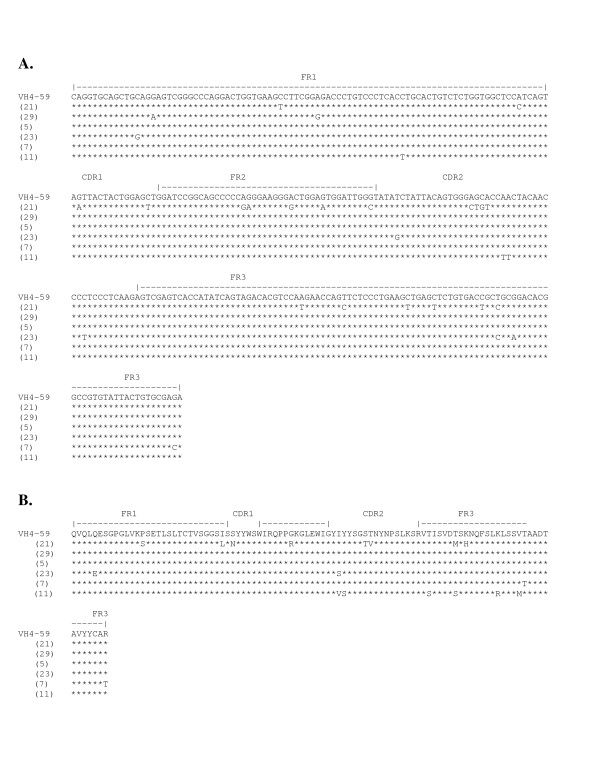
**Nucleotide sequences of the V_H _genes encoding xenoantibodies in the V_H_4 family**. The IGVH4-59 germline progenitor encodes a proportion of xenoantibodies in patients mounting active xenoantibody responses. Shown are the sequences of immunoglobulin genes encoding xenoantibodies in patients at day 10 after placement on a bioartificial liver containing porcine hepatocytes. **(A.) **The nucleic acid sequence of genes encoding xenoantibodies in the V_H_4 family are most closely related to the human VH4-59 germline gene.Stars indicate identities in nucleic acid sequence. Clone numbers are indicated in parenthesis. Nomenclature of the germline genes, alignments and numbering are based on the human immunoglobulin germline gene table [68]. **(B.) **Translated amino acid sequences of V_H_4 genes encoding xenoantibodies compared with the amino acid sequence of the closest human germline progenitor VH4-59.

We have used an anchor-ELISA PCR analysis to demonstrate that xenoantibodies are predominantly encoded by genes in the in the V_H_3 family and a smaller proportion of these antibodies are encoded by genes in the V_H_4 family [6,10]. The production of cDNA libraries from the peripheral blood of patients mounting active xenograft responses has allowed us to apply colony filter hybridization and nucleic acid sequencing to identify the germline progenitors encoding these antibodies. We identified the IGHV3-11 and IGHV3-74 germline genes as the progenitors of xenoantibodies produced by the V_H_3 family of immunoglobulin genes, however the progenitors encoding xenoantibodies in the V_H_4 family were not previously identified. To complete the structural analysis of immunoglobulin genes encoding xenoantibodies, we produced five V_H_4 cDNA libraries from peripheral blood samples obtained from patients mounting active xenoantibody responses to pig cells. The cDNA libraries were used to determine whether an increase in gene expression associated with any of the germline progenitors in the V_H_4 family could be identified when comparing Ig gene usage in patients prior to and following exposure to pig cells. On the basis of immunoglobulin gene sequencing of 48 genes in each library, the relative expression of the VH4-59 germline gene was increased from 5–8% at day 0 to 43–44% at day 10 post-xenoantigen exposure. The nucleotide and animo acid sequence of six of these genes is shown in Figure 1. The genes encoding IgM xenoantibodies in the V_H_4 family, similar to those encoding V_H_3 xenoantibodies, are expressed in germline configuration. We have previously reported that xenoantibodies encoded by the V_H_3 family are structurally-related and can be placed in one of seven structural classes of immunoglobulin genes [6]. Xenoantibodies in the V_H_4 family are characterized by a 1-1 canonical structure. This data indicates that the shape of the binding pocket of this small group of antibodies differs from that encoded by the IGHV3-11 germline genes.

### The DPK5 and HSIGKV134 germline genes are used to encode the light chain of xenoantibodies

**Figure 2:**
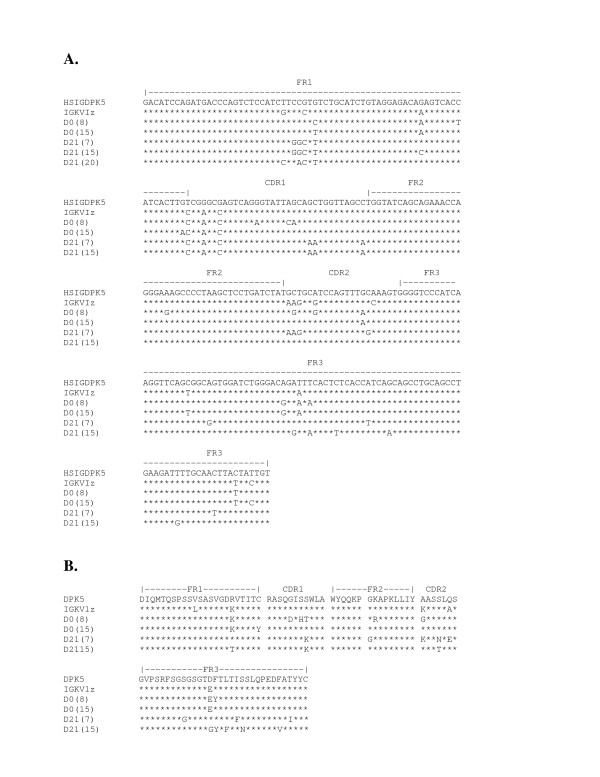
**The nucleotide and animo acid sequence of light chain genes encoding xenoantibodies induced in non-human primates. (A.)** The nucleotide sequence of genes encoding the light chain of xenoantibodies are most similar to the human HSIGDPK5 and HSIGKV134 germline genes. The sequences shown here are aligned with the human HSIGDPK5 germline gene as well as to the closest germline progenitor identified in non-human primates, IGKVIz. The sequence of the genes encoding the light chain of xenoantibodies at day 21 is compared with that at day 0. Clone numbers are indicated in parenthesis. **(B.)** The amino acid translation of these sequences is shown.

**Figure 3:**
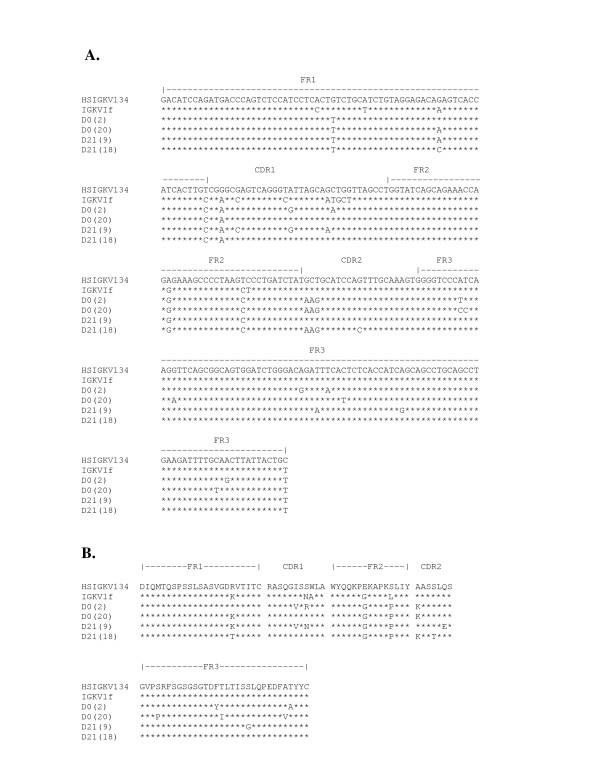
**The sequence of the light chain genes encoding xenoantibodies in non-human primates that are most similar to the human germline gene HSIGKV134. (A.)** The nucleotide sequence of genes encoding the light chain of xenoantibodies aligned with the human germline gene HSIGKV134 and the closest non-human primate germline gene, IGKVIf, is shown. The sequences of the genes encoding xenoantibody light chains at days 0 and 21 are compared. Clone numbers are indicated in parenthesis.**(B.)** The amino acid sequence of genes encoding the light chain of xenoantibodies.

In humans and non-human primates, a restricted group of genes encodes the heavy chain of xenoantibodies, but information on light chain genes is very limited. Due to insufficient sequence information, the determination as to whether a similar restriction in light chain gene usage occurs cannot be made, nor can accurate molecular models of xenoantibodies be generated. We have previously shown that xenoantibodies encoded by the IGHV3-11 IgV_H _germline progenitor can bind to the gal carbohydrate when paired with the DPK9 light chain gene [6]. In non-human primates, the light chain genes encoding xenoantibodies have not been defined. We therefore prepared cDNA libraries from peripheral blood samples of non-human primates mounting active xenoantibody responses following exposure to porcine hepatocytes or heart xenografts. Our earlier studies showed that the IGHV3-11 germline progenitor encodes xenoantibodies in these non-human primates [9,10], and the same samples were now used to identify the light chain genes expressed at this time. Serum samples were initially used to verify that an induced xenoantibody response could be documented by ELISA and cells were used to produce a series of cDNA libraries [10]. We prepared 6 cDNA libraries from rhesus monkeys prior to and at day 21 following exposure to pig cells to identify the germline progenitors encoding light chains. The relative frequency of Ig gene usage by individual germline progenitors was determined by sequencing 40 random clones from each library. The results indicate that xenoantibodies expressed at day 21 were encoded by a restricted number of germline progenitors. The closest matching human germline genes were DPK5 and HSIGKV134 in the VK I family (Figures 2 and 3 respectively). The closest matching germline sequences in the rhesus monkey were IgKV1f and IgKV1z which showed 95% and 93% nucleic acid sequence homology with the day 21 post-transplant rhesus monkey genes encoding xenoantibodies [28]. To date, little information is available on V_H _and V_L _germline genes in non-human primates. The rhesus germline light chain genes are members of the V kappa 1 subgroup and are of the same canonical structural class as human light chain genes encoding single chain antibodies that bind to the gal carbohydrate. The same germline progenitors were used to encode light chains in xenoantibodies induced following immunization with porcine hepatocytes or transplantation with porcine heart xenografts. This information was used in the computer-simulated model to complete our analysis of the binding site configuration in xenoantibodies selected for expansion in primates.

### Computer-simulated modeling of the xenoantibody/gal binding pocket

**Figure 4:**
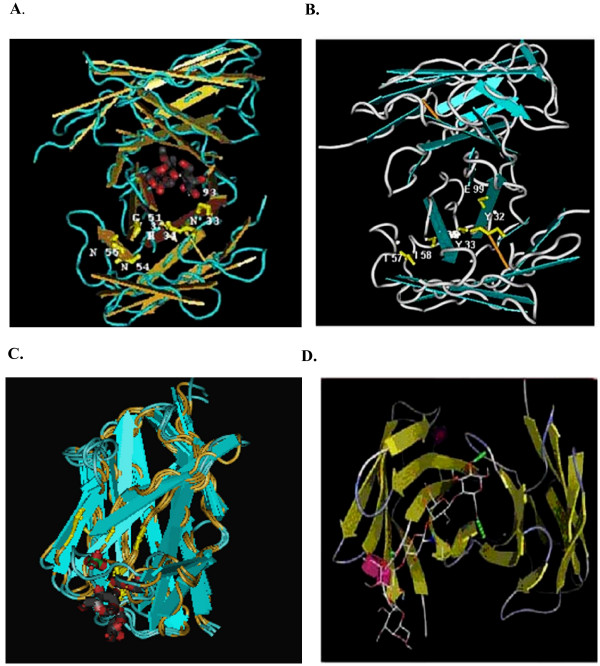
**Computer-simulated model of xenoantibody/carbohydrate interaction in humans and non-human primates. (A.)** Anti-carbohydrate single chain antibody (assession number IMFA) complexed with a trisaccharide carbohydrate antigen. Amino acids in key contact sites identified by crystallography are shown. The information can be accessed in the NCBI database by homology search for structural similarities. **(B.)** The IGHV3-11 xenoantibody (122B) induced in human patients mounting an active immune response following placement on a bioartificial liver.  **(C.)** Computer-simulated model of the IGHV3-11 human single chain xenoantibody (122B) aligned with the VH4-59 germline progenitor (clone 5, Fig 1). **(D.)** The IGHV3-11 V_H_ gene [9] and DPK5 light chain genes (clone 17, Fig 2) encode a xenoantibody expressed at high levels post-transplantation with porcine hearts and following immunization with porcine hepatocytes in rhesus monkeys.

**Figure 5:**
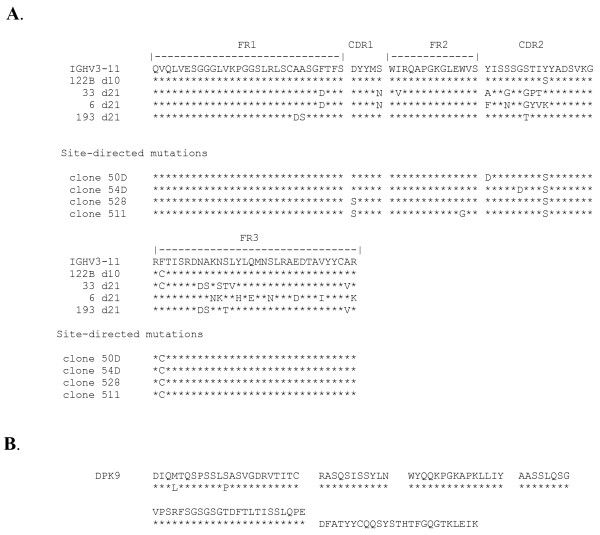
**Amino acid translation of the sequences of genes encoding human xenoantibodies. (A.)** The amino acid translation of immunoglobulin heavy chain genes in the V_H_3 family encoding xenoantibodies at days 10 and 21 following exposure to porcine hepatocytes.  The sequences at day 10 are expressed in germline configuration, however, the sequences at day 21 demonstrate the onset of mutations. Site-directed mutations were introduced into the germline gene in positions shown. **(B.)** The amino acid sequence of the genes encoding the light chain of a human xenoantibody that binds to the gal carbohydrate is shown compared with the closest germline progenitor, DPK9.

The structure of the binding site was addressed using computer-simulated molecular models generated by homology modeling (Figure 4). Computer-assisted homology-based modeling provides a reliable method of defining the three-dimensional structure of the binding pocket of xenoantibodies. Using this methodology, antibodies whose structure has been defined by crystallography can be used as a template for structural studies on an antibody of interest. Three-dimensional structural data obtained by modeling with homology-based techniques agrees well when compared with data determined by x-ray crystallographic techniques [32]. We used the AUTODOCK and DOCK programs for our model to identify contact sites relevant for xenoantibody binding to the gal carbohydrate (Arthur Olson, La Jolla, CA), [33,34]. Key contact sites relevant for optimal xenoantibody/gal carbohydrate interaction were identified by docking the gal carbohydrate in trisaccharide and pentasaccharide forms into the computer-simulated model of the IGHV3-11 xenoantibody. Energy scores were computed and the model selected on the basis of the lowest energy conformation. The results of the computer-assisted model indicate that the light chain, heavy chain and CDR3 region each contribute to gal saccharide binding. Key contact sites include positions 31and 33 of the CDR1, 50, 57 and 58 of the CDR2, and 99–103 of the CDR3. Positions 32 and 49 of the light chain also contribute to optimal binding to gal tri and pentasaccharide. Induced xenoantibodies and antibodies that demonstrate the ability to bind to carbohydrates have a structurally-similar binding pocket (Figure 4, [35,36]). Interestingly, mutations identified *in vivo *in IgG xenoantibodies expressed in patients undergoing active xenoantibody responses occur at several predicted contact sites (Table 1, Figure 5). Mutations located in positions 50, 57 and 58 in the heavy chain of IgG xenoantibodies were identified in both humans and non-human primates mounting active xenoantibody responses (Figure 5, [9,10]).

### The affinity of xenoantibodies for the gal carbohydrate can be altered by site-directed mutations

**Figure 6:**
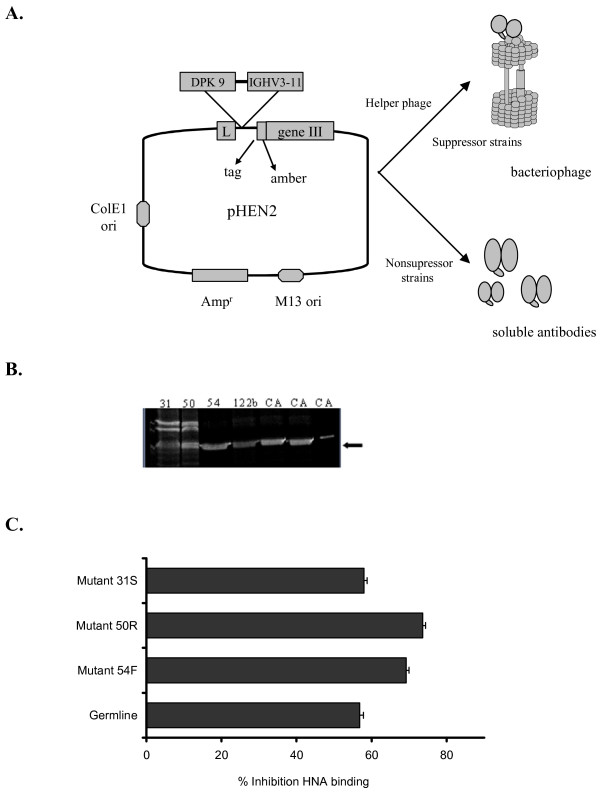
**Single chain xenoantibodies were produced using the pHEN vector and expressed as soluble antibodies.  **The xenoantibodies with site-directed mutations were compared with the germline progenitor for the ability to inhibit natural antibody binding. **(A.)** The IGHV3-11 gene encoding xenoantibodies in human patients was cloned into the vector pHEN2.  Single chain xenoantibodies were expressed as phagemid or soluble antibodies for functional studies. **(B.)** The soluble antibodies were run on an SDS page gel to confirm that antibody was produced. **(C.)** The ability of the soluble single chain antibodies with specific site-directed mutations to block human xenoantibody binding was compared by inhibition ELISA. The ability to bind more efficiently to purified gal carbohydrate, and thereby block human xenoantibody binding more effectively was compared in this assay.  Single chain antibodies with a mutation at site 50 block the binding of human xenoantibodies most effectively.

The CDR1 region, identified as important for xenoantibody/gal carbohydrate interaction in the molecular model, is identical in xenoantibodies induced in patients and non-human primates [5-8]. Interestingly, the CDR1 sequence of the anti-gal monoclonal antibody 22.121 that identifies the residual gal carbohydrate encoded by the iGb3S gene following knockout of the galactosyltransferase gene also utilizes this CDR1 region [7]. We selected this site for site-directed mutagenesis to create a mutation in position 31 (aspartic acid) in the xenoantibody encoded by IGHV3-11 to address whether the affinity of antigen/antibody interaction would be altered by this modification. The replacement of an aspartic acid with a serine residue was accomplished by overlap extension PCR. The gene was sequenced to confirm that the correct mutation was introduced in the desired location. Site-specific mutations were also introduced at sites 50 and 54 within the CDR2. Site 50 is frequently mutated in IgG xenoantibodies expressed in patients and in non-human primates (Figure 5, [6,9,10]). The ability of the mutated antibodies to bind to gal and block human natural antibody binding to purified gal pentasaccharide was measured using an ELISA assay. The results show that the introduction of a mutation in position 50 improved the ability of the single chain antibodies to block human natural antibody binding to purified gal, whereas mutations at other sites were less effective (Table 1, Figure 6).

### Binding affinity is not altered by somatic mutations in IgG genes encoding xenoantibodies in patients following BAL treatment

**Figure 7:**
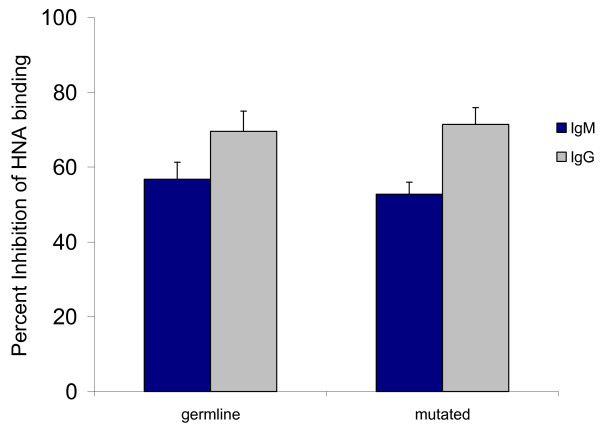
**Xenoantibodies expressed in germline and somatically-mutated configurations demonstrate comparable ability to block human natural IgM and IgC xenoantibody binding to pig cells. **Xenoantibodies cloned from the peripheral blood of human patients mounting active xenoantibody responses were expressed as single chain xenoantibodies. The ability of xenoantibodies with mutations (clone 33) to block human natural antibody binding to pig cells was compared with that of the antibody expressed in germline configuration (clone 122B) by flow cytometry. The antibody expressed in germline configuration was as efficient as the antibody expressed in somatically-mutated form in the ability to block xenoantibody/xenoantigen interaction.

IgM xenoantibodies directed at the gal carbohydrate in human patients treated with a bioartificial liver hepatic support device (BAL) containing pig cells are initially expressed in the absence of somatic mutation [6]. IgG xenoantibodies that are detected by day 21 demonstrate mutations in approximately 67% of the clones sequenced. These mutations were not localized to the CDR regions of the antibodies [6]. Sites at which mutations were identified *in vivo *in libraries of IgG clones encoding xenoantibodies at day 21 after xenoantigen exposure included positions 35, 50, 56, 57 and 58 (Figure 5). The amino acid substitutions resulting from these mutations included the substitution of a serine with an asparagine residue at position 35 (both uncharged and polar), a tyrosine for an alanine or a phenylalanine at position 50 (substitution of an uncharged polar side chain for a nonpolar side chain) and changes at position 56 and 57 for neutral amino acids which were either polar or non-polar. In order to study the biological role of the occurrence of mutations that were identified in antibodies produced in patients mounting active anti-gal xenoantibody responses to pig cells, the IgV_H _gene (IGHV3-11) in germline and somatically-mutated forms was cloned from the peripheral blood of patient samples and expressed as single chain xenoantibodies using the vector (pHEN2) (Table 2, Figure 6). The light chain gene used in these constructs was the DPκ9 germline progenitor (Figure 5). Single chain antibodies encoded by IGHV3-11 bind to the gal carbohydrate *in vitro *when expressed in germline configuration, indicating that specificity for this carbohydrate is inherent in the binding pocket formed by the IGHV3-11 heavy chain and DPk9 light chain. Soluble single chain xenoantibodies expressed in germline and somatically mutated forms were compared for their ability to bind to pig cells and block human natural antibody binding to cells of xenogeneic origin (Table 2, Figure 7). Preincubation with soluble antibodies results in inhibition of human natural IgM and IgG antibody binding to pig endothelial cells, ranging from 56–71%, as measured by flow cytometry. Germline and somatically-mutated antibodies demonstrated no significant differences in the ability of equivalent concentrations of soluble single chain antibodies to block human xenoantibody binding to pig cells (Table 2, Figure 7). The relative affinity of the germline form of the antibody for the purified gal carbohydrate in pentasaccharide form was 1.1 × 10^-8 ^M, compared with 2–4 × 10^-8 ^M when 14 amino acid substitutions were introduced during the evolution of the immune response of antibodies encoded by the IGHV3-11 germline progenitor (Figure 5, Table 2). The affinity for the gal carbohydrate remained in the 10^-8 ^M range despite the presence of mutations in genes encoding IgG xenoantibodies in these patients.

## Discussion and conclusion

Xenoantibodies that initiate the rejection of porcine xenografts are included under the broad classification of "natural antibodies" in humans [3,4]. Natural antibodies are believed to play a primary role in defense against pathogens before specific antibodies are produced by the immune system. For many years, natural antibodies were defined as polyreactive antibodies that bind to multiple target antigens such as nucleic acids, proteins and polysaccharides with low-to-moderate affinity [37-39]. More recently, high-affinity, monospecific natural antibodies have been identified that bind to a single target with kinetic constants similar to those observed for immune antibodies. Monospecific natural antibodies may be expressed in germline configuration [40], raising the intriguing possibility that the specificity of groups of natural antibodies is inherent in the structural configuration of the binding site [2,41].

Our laboratory has been studying the structure, sequence, and germline origin of xenoantibodies in several models of xenograft rejection. A small number of closely-related Ig genes that are evolutionarily conserved in structure encode xenoantibodies when expressed in germline configuration in all species studied to date [6,7,9,10,42,43]. The repertoire of pre-existing anti-gal antibodies that is present in normal individuals in the absence of immune stimulation, has been demonstrated in several experimental settings to include antibodies encoded by a relatively small number of germline progenitors. Knockout mice and patients mounting active immune responses to porcine xenografts demonstrate a selective expansion of anti-gal antibodies encoded by one or two germline progenitors from the available repertoire [6-10]. The molecular basis for the expansion of xenoantibodies encoded by selected germline progenitors from a larger repertoire of pre-existing natural xenoantibodies with the ability to bind to the gal carbohydrate is not understood. Clarification of the structural requirements for optimal xenoantibody gal/carbohydrate interaction may provide insight into the process by which xenoantibodies that mediate the rejection of porcine xenografts are recruited. Furthermore, this information may be directly used to develop new strategies or optimize existing strategies for preventing xenoantibody/carbohydrate interaction [44-46].

In humans, anti-gal xenoantibodies are predominantly encoded by a small group of V_H_3 genes [6,7]. Antibodies selected for expansion in patients mounting anti-gal responses can be classified into one of the seven canonical structural groups for IgV_H _genes [29,30], and display a well-defined structural conformation of the binding pocket. The evolutionary conservation of antibodies with this structure suggests they play a role in host defense [31,41,47]. The presence of the gal carbohydrate on certain types of enteric bacteria provides a basis for the selection, retention, and redundance in the ability of the immune system to initiate an antibody response with this defined specificity [47]. We have established that somatic mutation is not required for xenoantibody binding to the gal carbohydrate [6]. IgG xenoantibodies induced in humans at day 21 post-BAL display mutations that are neither localized in the CDR regions of the xenoantibodies nor do they significantly enhance the binding of xenoantibodies to purified gal pentasaccharide [48,6]. Mutations do not appear in key sites that determine the canonical structure of anti-gal antibodies, or if they do occur, are not selected for in xenoantibodies expanded in response to pig cells [6]. This data is consistent with the findings of Dehoux et al. who demonstrate that no significant difference in the affinity of natural antibodies that mediate hyperacute rejection and those that initiate acute vascular rejection of porcine kidney xenografts could be demonstrated [23]. In these baboons, an isotype switch occurs that is associated with an increase in all IgG subclasses, especially IgG1 but no increase in the affinity of IgG xenoantibodies for the gal carbohydrate. Similarly, Cotterell et al have reported that the avidity of human IgM xenoantibodies before and after perfusion through porcine livers remains unchanged [49]. The affinity of xenoantibodies that bind to the gal carbohydrate in baboons and humans is generally between 1–4 × 10^-8 ^for IgM and IgG xenoantibodies, but may vary within the range of 10^-7 ^to 10^-10 ^M as reported by several groups [5,21,23]. Antibodies produced in humans transplanted with porcine islet cells demonstrate an increase in affinity for the gal carbohydrate from 2 × 10^-7 ^M to 1 × 10^-8 ^M, suggesting that an affinity maturation response occurred in these patients over several months time [22]. Affinity maturation may therefore not be evident in several settings until sometime later than 3 weeks post-transplantation [9-11], however in the interim, acute vascular rejection can occur. Since affinity of natural anti-gal xenoantibodies selected for expansion in human patients post-transplantation is relatively high when expressed in germline configuration in naïve individuals, our data suggests that polyreactive xenoantibodies with binding affinities within the order of 10^-3 ^to 10^-6 ^are not relevant for xenograft reactions.

On the basis of this information, and sequence data obtained from multiple models of xenograft rejection that have been studied in our laboratory, we have formulated the hypothesis that a unique structural configuration (defined by the canonical structure of the immunoglobulin light chain, heavy chain and CDR3 region) defines IgM and IgG xenoantibodies directed at pig cells and/or xenografts in the presence or absence of somatic mutation [6]. The concept that the shape of an antigen binding site is specialized to conform to specific types of antigen/antibody interactions, in general, was originally proposed by Lara-Ochoa et al. [41]. Interestingly, recent studies have indicated that although a potentially large combination of canonical structures is possible, only a few are selected and utilized in the human antibody repertoire [31]. Specifically, out of 300 possible canonical structural combinations, only ten are preferentially used. The process of molecular recognition, therefore, may begin with a basic recognition of an antigen with a defined shape by small numbers of germline progenitors with a specific main chain structural conformation. This first stage of antigen/antibody recognition, proposed to be the basis for the evolutionary selection of small groups of structurally-related antibodies, may provide an explanation for the well-documented restriction in immunoglobulin genes encoding xenoantibodies. Antigen/antibody interaction initiated by binding to antibodies with a select main-chain conformation, followed by a second stage of selection for antibodies with specific amino acids in key contact sites within the hypervariable region sequence that are relevant for optimal for xenoantibody/gal interaction is a concept consistent with our data. In humans, anti-gal antibodies in unimmunized individuals are encoded by a group of 6–8 well-defined and structurally-related germline progenitors [5]. Exposure to pig cells in humans, monkeys or mice results in the selective expansion of xenoantibodies encoded by one or two of these germline genes [6-10]. The basis for this second stage of selection may be clarified by a detailed analysis of the structure of the xenoantibody/gal binding site [19]. Amino acids in specific contact sites defined by computer-simulated models may play a role in the selection of antibodies for expansion. In other types of natural antibody/antigen interactions, such as binding to single-stranded DNA and insulin, single amino acid mutations have the ability to significantly increase the affinity and/or alter the specificity of the antibody [40]. Seemingly minor changes in amino acids located in key contact sites relevant for xenoantibody/gal carbohydrate interaction may dramatically modify natural antibody responses. The identification of key contact sites and the application of this information to understanding the role of mutations in xenoantibodies that mediate the rejection of porcine xenografts would provide a solution to the controversy regarding the functional role of mutations in delayed xenograft rejection.

Although contact sites are best defined by crystallography, computer-simulated modeling combined with site-directed mutagenesis has been used successfully to define the sites critical for antibody/antigen interaction [32,50]. The construction of engineered antibodies and three-dimensional models representing antigen/antibody interaction defines the binding site of each unique antigen/antibody interaction in a way that cannot be done using sequence information alone. At the three-dimensional level, the unique folding of CDR and framework segments mutually influence each other's conformation. The structural repertoire of the CDR is characterized by a finite number of main chain conformations, yet the differences in key amino acids within the CDR contribute to the generation of a large number of antibodies with varied topologies. The wide diversity in antigen binding specificities is attributed largely, but not exclusively, to variation in surface features of the CDR [51-53]. Antigen binding involves multiple non-covalent interactions between atoms in the antigen and the CDR surface, although only about one third to one fifth of the CDR surface participates directly in antigen contact [54]. The generation of computer-simulated models provides a method to identify specific contact sites unique to each antibody/antigen interaction. The information gathered from these models is broadly applicable in antibody engineering, antigen docking and in structure-based drug design.

We have applied computer-simulated techniques to create a model of xenoantibody/gal carbohydrate interaction. The model has identified xenoantibody/gal pentasaccharide binding sites at positions 32 and 49 of the light chain, 31 and 33 of the CDR1, 50, 57 and 58 of the CDR2 and 99–103 within the CDR3 region of the heavy chain. Site-directed mutagenesis at a single site was demonstrated to alter the affinity of xenoantibody binding to the gal carbohydrate, as suggested by the model, however, the affinity of the xenoantibodies remain in the 10^-8 ^M range in the presence or absence of mutations *in vivo*. Functional analysis of anti-gal xenoantibodies cloned from cDNA libraries prepared from the peripheral blood of patients mounting active xenoantibody responses indicates that high affinity xenoantibodies are not produced *in vivo *in patients at day 21 following at least two exposures to pig cells in the bioartificial liver hepatic support device. Although this could be attributable to the method and/or length of exposure of these patients to porcine cells, it is equally possible that somatic mutation does not contribute to any appreciable increase in binding affinity for antibodies that mediate the xenograft rejection response. Precedent for the latter possibility has been shown to occur in other types of antibodies that play a key role in rapid defense responses, such as protective antibodies produced in response to viruses and antibodies that mediate human immune responses to Haemophilus influenzae b capsular polysaccharide [55,56]. Antibodies directed at pathogenic agents with repetitive antigenic determinants demonstrate high affinity binding determined exclusively by specific germline V_H _and V_L _combinations in the absence of mutations and have similar binding affinities when expressed in germline and somatically-mutated configurations [57]. Ongoing pressure therefore exists to conserve the germline configuration of the variable region of these antibodies [58]. Our studies indicate that xenoantibodies respond in a manner that is very similar to other evolutionarily-selected antibodies that play a role in innate defense.

Contact sites for carbohydrate/xenoantibody interaction predicted by molecular models include amino acids within the third complementarity determining region of the heavy chain. The CDR3 is characterized by a large diversity in amino acid composition and length. Classification of these loops into canonical forms has been difficult due to this large variation in composition [52,53]. To date, thirteen canonical forms have been defined for the CDR3 region of immunoglobulin heavy chains. These forms represent a combination of a specific take-off geometry and specific apex, and are classified according to sequence motif, intra-loop interaction and/or main chain geometry. Although the loop take-off angle and specific apex geometry are important for defining the structure of the CDR3 segment, the plasticity of the antibody response ensures that select conformational structures may be achieved by multiple mechanisms. Our sequence information and molecular modeling data suggests that the CDR3 of xenoantibodies induced in response to exposure to pig cells is restricted in structure and contributes to antibody specificity. Molecular modeling of the IGHV3-11 antibody docked to the gal carbohydrate indicates that amino acids in positions 99–103 (EYLSS) are important for xenoantibody/gal interaction. Consistent with most antigen-antibody interactions, contacts at the end of the loop closer to the center of the combining site contribute to the specificity of xenoantibody/gal interactions. In humans and non-human primates mounting active xenoantibody responses, this CDR3 is selectively utilized in xenoantibodies that are expanded in response to porcine hepatocyte, heart and islet transplantation [6,9,10]. In patients, V_H _and V_L _germline genes encoding antibodies with a specific light chain, heavy chain and CDR3 are recruited in response to pig cell exposure. Although this pattern of antibody selection may be driven by a specific type of xenoantigen presentation and/or the time of exposure to porcine xenoantigens, a similar pattern of selection has been defined in knockout mice exposed to solid organ xenografts [8]. This restriction suggests that antibodies binding to carbohydrates are further selected on the basis of usage of specific CDR3 and J regions.

The significance of the xenoantibodies encoded by the V_H_4-59 germline progenitor has yet to be determined. Although xenoantibodies encoded by the IGHV3-11 germline progenitor encode most of the induced xenoantibodies in humans and non-human primates mounting active xenoantibody responses to xenografts from wild-type pigs, the minor population of xenoantibodies encoded by the V_H_4 family could bind to non-gal xenoantigens. The determination as to whether these IgV_H _genes are induced during delayed rejection of gal knockout organs would provide insight as to whether xenoantibodies directed at structurally-similar carbohydrates may play a role in delayed rejection. The role of this group of antibodies and the contribution of the complex CDR3 topography to binding affinity and specificity are the subject of ongoing studies in our laboratory.

## Methods

### RNA isolation

Total RNA was extracted from the peripheral blood lymphocytes obtained from human patients at days 0, 10 and 21 post-exposure to pig hepatocytes following placement on a bioartificial liver [59] and from rhesus monkeys at day 0 and 21 post transplantation with porcine heart xenografts or post-immunization with porcine hepatocytes [9,10]. A strong IgM and IgG xenoantibody response to pig endothelium and to the α-gal epitope was verified by ELISA [6,9,10]. All procedures in non-human primates were reviewed and approved by the Institutional Animal Care and Use Committee at the University of California at Davis. RNA extraction was performed using a solution containing 4 M GuSCN, 1.5 M Na citrate (pH 7.0) and 0.5% sarcosyl. RNA was subjected to a phenol/chloroform/isoamyl alcohol extraction, precipitated with isopropanol, then resuspended in 1 mM EDTA pH 8.0 and precipitated with 0.3 M sodium acetate and ethanol.

### PCR for analysis of the immunoglobulin V_H _genes encoding xenoantibodies

Total RNA isolated from PBL derived from patients prior to and following exposure to a bioartificial liver and non-human primates immunized with porcine hepatocytes and endothelial cells was used to prepare first strand cDNA using a μ-chain-specific primer (GGGAAAAGGGTTGGGGCGGATGCA) or γ-chain specific primers (GACCGATGGGCCCTTGGTGGA for human) and (GGGTTGTAGTCCTTGACCAGGCAG for monkeys). The cDNA/RNA hybrids were hydrolyzed with NaOH. A nested PCR reaction was used to clone and analyze the IgV_H _and IgV_L _genes that encode xenoantibodies [6]. The framework primers used to clone IgM antibodies from the IgV_H _3 and 4 families were (GAGGTGCAGCTGGTGGAGTCTGG and CAGGTGCAGCTGCAGGAGTC), respectively. Immunoglobulin gene products amplified in the first PCR reaction using Cμ and anchor primers were subjected to a nested reaction using Cμ primer #3 and a V_H_3 or V_H_4 gene family-specific primer containing an SfiI restriction site at the 5' end. The PCR was performed in a Perkin-Elmer 9600 GeneAmp PCR System Thermocycler for 1 cycle of 94°C 5 min., 30 cycles of 94°C for 45 seconds, 56°C for 60 seconds, 72°C for 90 seconds, and one cycle of 72°C for 7 minutes. The primers used to amplify immunoglobulin kappa light chain genes were kappa 4 (ACAGATGGTGCAGCCACAG) and kappa 5 (CAGATGGCGGGAAGATGAAG) which align with the Cκ gene and primers KJ04 (ATTGTAATGACACAG) and KJ05 (TCTGCATCTGTAGGAGAC) which align with the Framework 1 region of the light chain. The PCR products were verified by size on a 1.4% agarose gel and cloned into the TA 2.1 vector (Invitrogen, San Diego, CA). The cloned DNA was transformed into E. coli, and recombinant colonies identified for plasmid preparation on the basis of color screening. PCR products were ligated into the pCR 2.1 vector (Invitrogen, Carlsbad, CA) and transformed into INVαF' cells. A minimum of 40 colonies from each library were sequenced to establish the IgV_H _genes encoding antibodies by specific germline progenitors.

### Expression of the cDNA clones as single chain antibodies

Immunoglobulin genes encoded by the IGHV3-11 germline progenitor display an increased frequency of expression in B cells isolated from the humans and non-human primates mounting active xenoantibody responses. These genes, expressed in germline and somatically-mutated configurations were cloned into the single chain antibody expression vector (pHEN2, Center for Protein Engineering, Medical Research Council Centre, Cambridge, England). Single chain antibodies were used in flow cytometry experiments and ELISA to measure binding specificity for purified α-gal carbohydrate and for pig endothelial cells. Briefly, cloning was accomplished by amplification of the IGHV3-11 cDNA clones in a PCR reaction using the primers V_H_3 Back Sfi (GTCCTCGCAACTGCGGCCCAGCCGGCCATGGCCCAGGTGCAGCTGGTGGAG), V_H_3 Back LD3 (CAGGTGCAGCTGGTGGAGTCTGGGGGAGGCTTGGTC), J_H_3Xho (TCGACCTCGAGTTGAAGAGACGGTGACCATTGTCCCTTGGCCCCAGATATCAAAAGCATCCAAACTACTCAGGTACTCTCGC), J_H_3 IBA(GATATCAAAAGCATCCAAACTACTCAGGTACTCTCGC), and J_H_3 2 (TGAAGAGACGGTGACCATTGTCCCTTGGCCCCAGATATCAAAAGCAT). The PCR products were gel purified and ligated into the pHEN2 vector which had been restricted with the enzymes Sfi I and Xho I. The ligation was transformed into competent bacteria and screened using a V_H_3 primer and J_H_3 primer to check for the insert. Phagemid containing the cloned genes were grown in 2XTY containing 100 μg/ml ampicillin and 1% glucose and were infected with VCSM13 helper phage (Strategene Cloning Systems, La Jolla, CA) at a ratio of 1:20 (number of bacterial cells:helper phage). The infected cells were then grown in 2XTY containing 100 ug/ml ampicillin and 25 ug/ml kanamycin overnight at 30°C. The phage were precipitated with polyethylene glycol 6000/NaCl (20% PEG, 2.5 M NaCl), titered, and resuspended in PBS to 10^13 ^transducing units/ml. Nucleic acid sequencing was used to confirm that no additional nucleic acid substitutions were introduced into the cDNA clones during the PCR reaction used to modify the ends of the clones for compatibility with the phagemid vector. 10^9^–10^12 ^phage were used in ELISA assays to measure binding specificity for the gal carbohydrate [6].

### Screening phage particles by ELISA

The binding of phage particles expressing the IGHV3-11 germline gene as a single chain antibody to mouse laminin was addressed using Falcon Microtest III flexible assay plates (Becton Dickinson, Oxnard, CA) coated with target antigen at a concentration of 20 ug/well in PBS. The plates were coated overnight at room temperature, rinsed, blocked with 1% BSA for 2 hours at 37°C, incubated with 10^9^–10^12 ^phage particles for 90 minutes at room temperature, washed and incubated with HRP anti-M13 monoclonal conjugate (Pharmacia Biotech, Piscataway, NJ) at a concentration of 1:5000 for 30 minutes at room temperature. The reaction was developed with substrate solution (100 ul/well TMB (KPL, Gaithersburg, MD) in 100 mM sodium acetate, pH 6.0. Fifty μl of 0.18 M sulphuric acid was used to stop the reaction, and the OD was read at 650 nm and 450 nm using a Molecular Devices microplate reader.

### Expression of single chain antibodies in soluble form

The pHEN clones were infected into HB2151 and induced with IPTG to yield soluble antibody fragments that were identified on SDS polyacrylamide gels [6]. Eluted phage (10^5 ^transforming units) were infected into 200 ul of exponentially growing HB2151 bacteria for 30 minutes at 37°C, and plated on TYE plates with 100 ug/ml ampicillin and 1% glucose. Single colonies were grown to log phase in 2XTY containing ampicillin and 0.1% glucose and induced with IPTG at a final concentration of 1 mM. Bacteria were grown overnight at 30°C, spun at 1800 × g for 10 minutes, resuspended and quantitated using BIORAD Quantity One Software, Version 4.0.3 (BIORAD, Hercules, CA). Soluble single chain antibodies were used in inhibition ELISA assays to measure binding affinity and flow cytometry assays to measure the ability of these clones to block human xenoantibody binding to pig endothelial cells [60,61].

### Flow cytometry

Flow cytometric analysis was conducted to measure the ability of soluble single chain antibodies to block xenoantibody binding to pig xenoantigens on endothelial cells. Pig cells (1 × 10^6^) were preincubated (blocked) with 500 ng of soluble single chain antibodies in germline or mutated configurations for one hour at 4°C. These samples were then incubated with serum samples obtained from normal humans at a dilution of 1/20 for 30 minutes at 4°C. The cells were then washed and incubated with 100 ul of FITC-conjugated mouse anti-human IgG or IgM at a 10 ng/ul dilution (Serotec, Cambridge, England) to detect binding of human natural antibodies to pig endothelial cells [61]. Binding of the IB4 lectin (100 ul of a 1:100 dilution, 1 mg/ml stock) was used to detect levels of the α-gal epitope expressed on pig cells in the presence or absence of scFv fragments. Following three washes, fluorescence was read on a FACScan (Becton Dickinson).

### Flow cytometric cytotoxicity assay

This assay measures antibody-mediated, complement-dependent cytotoxicity and was used to determine whether blocking of human natural antibody binding to pig cells was associated with a reduction and/or elimination of complement-mediated cytoxocity [62]. PAEC were incubated with human serum at a 1/20 dilution for 30 minutes, followed by the addition of rabbit complement to each tube and incubation for one hour. Propidium iodide was added to the samples which were incubated for 5 minutes, then washed with PBS containing 0.2% azide. The samples were then ready to be acquired by the FACScan. The uptake of propidium iodide was indicative of cell death.

### Site-directed mutagenesis

Site-directed mutagenesis was done by overlap extension PCR [63]. The 71A immunoglobulin gene encoding the xenoreactive IGHV3-11 antibody in post-transplant samples was used as template DNA. The primers JH3XH01 and VH3BACKSfi, 50 forward (TTTCAGACATTAGTAGTAGTGGT) and 50 reverse (ACCACTACTACTAATGTCTGAAA) were used to allow the substitution of Y (Tyrosine) with D (aspartic acid) in the CDR2 region of the xenoantibody. The CDR1 mutation was introduced using the primers 31 forward (TTAGTAGCTACTACATGAGCTGG) and 31 reverse (CCAGCTCATGTAGTAGCTACTAA). This allows the substitution of an S (Serine) in place of D (Aspartic acid) in position 31. The PCR conditions included one cycle of 94°C for five minutes and 30 cycles of 94°C for 30 sec, 60°C for 30 sec and 72°C for 30 sec, then one cycle of 72°C for seven minutes. PCR products were run on an agarose gel to confirm the presence of a product of correct molecular weight.

### ELISA

Soluble single chain antibodies were tested by ELISA for binding to purified gal di, tri and pentasaccharide (Dextra Laboratories, UK) [60,61]. ELISA plates (MAXIsorb, Nunc) were coated overnight at 4°C with 50 ul of purified gal saccharide at a concentration of 5 ug/ml in bicarbonate buffer pH 9.6. Plates were washed and blocked with 0.5% Tween 20 in PBS for 1 hour prior to the addition of single chain antibodies in concentrations ranging from 0.1–100 ng/well. Single chain antibodies were incubated with the saccharide for 1.5 hours prior to washing and the addition of anti-hexahistidine antibody at a dilution of 1/500 for 30 minutes. Plates were washed 3 times, substrate was added followed by stop solution, and plates were read at 405 nm.

### Inhibition ELISA

Plates were precoated with gal pentasaccharide and blocked as described above. Dilutions of single chain antibodies demonstrated to result in 75% of maximum OD were mixed with soluble antigen (purified gal pentasaccharide) at concentrations ranging from 0.3–300 ug/ml overnight at 4°C prior to transferring the mixture to the antigen-coated ELISA plate [64]. The mixture was incubated for 2 hours at room temperature prior to the addition of human natural antibodies obtained from normal human serum at a concentration of 1/20. The plates incubated with human natural antibodies were washed and peroxidase conjugated goat anti-human IgG at a dilution of 1/5000 or goat anti-human total Ig at a dilution of 1/2500 was added. Plates were washed, developed and read at 405 nm.

### Statistics

The experiments involving antibody binding studies were repeated a minimum of three times, and samples were run in duplicate for each experimental timepoint. The data presented include the standard deviation of the mean.

### DNA sequencing

Single chain antibody clones and immunoglobulin gene clones were sequenced using the ALFexpress automated DNA sequencer and the Autocycle sequencing kit (Pharmacia Biotech, Alameda, CA). pHEN vector-specific primers FOR Link Seq(GCC ACC TCC GCC TGA ACC) and pHEN-SEQ (CTA TGC GGC CCC ATTCA) were used to sequence the heavy and light chains cloned into these vectors. Immmunoglobulin gene clones were sequenced using the M13 universal primer 5'(cy5) GTAAAACGACGGCCAGT-3' and M13 reverse primer 5'(cy5) AACAGCTATGACCATG-3.'

The closest germline genes were identified by BLAST [65].

### Molecular modeling

Swiss-Model and Predict Protein are internet-based tools for automated comparative protein modeling. They were used to run a homology model search [66] to find a heavy chain model with a structure similar to IGHV3-11 that has been established by x-ray diffraction. The 1DEE PDB file entry is an IgM heavy chain that is 90% similar to the human xenoantibody cloned in our laboratory that was used for the computer-simulated model. This structure was selected because backbone structural error was not introduced by insertions or deletions of amino acids using this antibody as a template for the IGHV3-11 heavy chain model. The IGHV3-11 heavy chain model was created using the IDEE antibody as a template and Hyperchem software (Hypercube, Boulder, CO) for the generation of the homology model. The sequence/structure compatibility of the xenoantibody model with the 1DEE sequence and crystallographic structure was assessed using three dimensional profile analysis. The profile comparison indicated a high level of overall compatibility of the amino acid sequence with its modeled three dimensional structure, and the absence of significant local ambiguities. The crystal water molecules generated from coordinates identified in 1DEE were placed in the corresponding regions of the xenoantibody model. Energy minimization was used to relax the contacts. We created models of xenoantibody/gal interaction (gal di, tri and pentasaccharide) using DOCK 4.0 software[67] and Autodock (Arthur Olson, Molecular Graphics Lab, La Jolla, CA) for geometric docking simulation [33]. Select amino acids scored as key contact sites using this model were modified using site-directed mutagenesis *in vitro *to test the validity of the models.

### Genebank listing

Nucleic acid sequences have been deposited in Genebank and are listed as assession numbers [Genbank: AY994517–AY994523] for the light chain sequences and [Genbank: AY986385–AY986390] for the V_H_4 sequences.

## List of abbreviations

α-gal epitope (Gal α1-3Galβ1-4GlcNAc-R)

## Authors' contributions

MKJ prepared the cDNA libraries, cloned and sequenced the single chain xenoantibodies, contributed to the experimental design and wrote the manuscript, NB and MY performed the affinity binding studies, RM prepared the computer-simulated models and identified xenoantibody/gal carbohydrate contact sites, NH and AX performed site-directed mutagenesis and contributed to the binding affinity studies, IS contributed to the generation of the computer-simulated models and sequenced the light chain genes, DVC edited the manuscript and contributed to the experimental design. The authors have had the opportunity to both read and revise the manuscript.

## Figures and Tables

**Table 1:** ScFv xenoantibodies constructed to study the role of mutations in xenoantibody/gal carbohydrate interaction

ScFv Antibody Clone	Location of Mutations in Amino Acids	Source of cDNA
122B IGHV3-11 patient d10 germline clone	58, 67	IgM human xenoantibody clone isolated at day 10
33 (IGHV3-11 with mutations)	28,35,37,50,52a,55,56,57 73, 74, 76, 77, 78, 94	IgG human xenoantibody clone isolated at day 21
193 (IGHV3-11 with mutations)	23,24,55,67,73,74,77,93	IgG human xenoantibody clone isolated at day 21
511 (IGHV3-11 site-directed mutation)	31,47	Site-directed mutagenesis of germline IGHV3-11 clone in germline configuration
528 (IGHV3-11 site-directed mutation)	31	Site-directed mutagenesis to modify amino acid #31 in the IGHV3-11 clone expressed in patients at day 10.
50D (IGHV3-11 site-directed mutation)	50	Site-directed mutagenesis to modify amino acid #50 in the IGHV3-11 clone expressed in patients at day 10.
54D (IGHV3-11 site-directed mutation)	54	Site-directed mutagenesis to modify amino acid #54 in the IGHV3-11 clone expressed in patients at day 10.

**Table 2:** Percent inhibition of natural antibody binding to PAEC with ScFv antibodies

ScFv Antibody Clone	Percent inhibition of IgM natural antibody binding	Percent inhibition of IgG natural antibody binding	Percent decrease in complement-mediated cytotoxicity to PAEC	Binding affinity (M) of xenoantibodies for gal saccharide
germline d10	56.8 +/- 4.5	69.6 +/- 5.4	20.4 +/- 4.4	1.1 – 1.6 × 10^-8^
mutated d21	52.8 +/- 3.2	71.4 +/- 4.5	18.5 +/- 5.5	2.3 – 4.2 × 10^-8^
